# Addition of probenecid to oral β-lactam antibiotics: a systematic review and meta-analysis

**DOI:** 10.1093/jac/dkac200

**Published:** 2022-06-21

**Authors:** Richard C Wilson, Paul Arkell, Alaa Riezk, Mark Gilchrist, Graham Wheeler, William Hope, Alison H Holmes, Timothy M Rawson

**Affiliations:** National Institute for Health and Care Research Health Protection Research Unit in Healthcare Associated Infections and Antimicrobial Resistance, Imperial College London, Hammersmith Campus, Du Cane Road, London W12 0NN, UK; Centre for Antimicrobial Optimisation, Imperial College London, Hammersmith Hospital, Du Cane Road, Acton, London W12 0NN, UK; Imperial College Healthcare NHS Trust, Hammersmith Hospital, Du Cane Road, London W12 0HS, UK; Centre for Antimicrobial Optimisation, Imperial College London, Hammersmith Hospital, Du Cane Road, Acton, London W12 0NN, UK; Imperial College Healthcare NHS Trust, Hammersmith Hospital, Du Cane Road, London W12 0HS, UK; Centre for Antimicrobial Optimisation, Imperial College London, Hammersmith Hospital, Du Cane Road, Acton, London W12 0NN, UK; National Institute for Health and Care Research Health Protection Research Unit in Healthcare Associated Infections and Antimicrobial Resistance, Imperial College London, Hammersmith Campus, Du Cane Road, London W12 0NN, UK; Centre for Antimicrobial Optimisation, Imperial College London, Hammersmith Hospital, Du Cane Road, Acton, London W12 0NN, UK; Imperial College Healthcare NHS Trust, Hammersmith Hospital, Du Cane Road, London W12 0HS, UK; Imperial Clinical Trials Unit, Imperial College London, Stadium House, Wood Lane, London W12 7RH, UK; Centre for Excellence in Infectious Diseases Research (CEIDR), University of Liverpool, Liverpool L7 8TX, UK; National Institute for Health and Care Research Health Protection Research Unit in Healthcare Associated Infections and Antimicrobial Resistance, Imperial College London, Hammersmith Campus, Du Cane Road, London W12 0NN, UK; Centre for Antimicrobial Optimisation, Imperial College London, Hammersmith Hospital, Du Cane Road, Acton, London W12 0NN, UK; Imperial College Healthcare NHS Trust, Hammersmith Hospital, Du Cane Road, London W12 0HS, UK; National Institute for Health and Care Research Health Protection Research Unit in Healthcare Associated Infections and Antimicrobial Resistance, Imperial College London, Hammersmith Campus, Du Cane Road, London W12 0NN, UK; Centre for Antimicrobial Optimisation, Imperial College London, Hammersmith Hospital, Du Cane Road, Acton, London W12 0NN, UK; Imperial College Healthcare NHS Trust, Hammersmith Hospital, Du Cane Road, London W12 0HS, UK

## Abstract

**Objectives:**

To explore the literature comparing the pharmacokinetic and clinical outcomes from adding probenecid to oral β-lactams.

**Methods:**

Medline and EMBASE were searched from inception to December 2021 for all English language studies comparing the addition of probenecid (intervention) with an oral β-lactam [flucloxacillin, penicillin V, amoxicillin (± clavulanate), cefalexin, cefuroxime axetil] alone (comparator). ROBINS-I and ROB-2 tools were used. Data on antibiotic therapy, infection diagnosis, primary and secondary outcomes relating to pharmacokinetics and clinical outcomes, plus adverse events were extracted and reported descriptively. For a subset of studies comparing treatment failure between probenecid and control groups, meta-analysis was performed.

**Results:**

Overall, 18/295 (6%) screened abstracts were included. Populations, methodology and outcome data were heterogeneous. Common populations included healthy volunteers (9/18; 50%) and those with gonococcal infection (6/18; 33%). Most studies were crossover trials (11/18; 61%) or parallel-arm randomized trials (4/18; 22%). Where pharmacokinetic analyses were performed, addition of probenecid to oral β-lactams increased total AUC (7/7; 100%), *C*_max_ (5/8; 63%) and serum *t*_½_ (6/8; 75%). Probenecid improved PTA (2/2; 100%). Meta-analysis of 3105 (2258 intervention, 847 control) patients treated for gonococcal disease demonstrated a relative risk of treatment failure in the random-effects model of 0.33 (95% CI 0.20–0.55; *I*^2 ^= 7%), favouring probenecid.

**Conclusions:**

Probenecid-boosted β-lactam therapy is associated with improved outcomes in gonococcal disease. Pharmacokinetic data suggest that probenecid-boosted oral β-lactam therapy may have a broader application, but appropriately powered mechanistic and efficacy studies are required.

## Introduction

Probenecid, p-(di-*n*-propylsulphamyl)-benzoic acid, was developed in 1949 with the purpose of decreasing the renal clearance of penicillin.^1^ Its mechanism of action is through competitive inhibition of organic anion transporters, which are responsible for excretion of organic agents, such as penicillin.^2^ Reduction in renal clearance of penicillin with probenecid demonstrated significant increases in serum exposure, meaning that lower doses of drug were required for similar pharmacokinetic/pharmacodynamic (PK/PD) target attainment. Probenecid’s influence on penicillin clearance became mainly academic in the post-war era as the capability to produce more diverse, cheaper and safer β-lactam antibiotics rapidly expanded.^1^ Probenecid remains a recommended adjunct in the management of some sexually transmitted infections to support therapeutic target attainment in compartments, such as CSF in neurosyphilis.^3^ However, its potential important and broader role in preserving the effectiveness of β-lactams through the optimization of β-lactam PK and dosing schedules needs to be considered, as well as possible adverse events associated with its use, such as nausea and unfavourable drug–drug interactions.

Globally, the WHO *Access*, *Watch* and *Reserve* (AWaRe) criteria require narrow-spectrum antimicrobials, such as the penicillins, to be available in appropriate type, dose and duration to treat common infections.^4^ With increasing drug resistance within common causative organisms, such as in streptococci, new methods to optimize the delivery of *Access* agents and protect the use of broader *Watch* and *Reserve* antimicrobials are required.^4,5^

It is not always possible to administer higher doses of an oral antibiotic to achieve an optimal PK/PD profile. In some instances, oral drug absorption or gastrointestinal side effects are associated with high doses and limit escalation of therapy. In other situations, augmented renal clearance may make achieving optimal drug exposure difficult. Some agents are not licensed for use at oral doses that would be required to obtain acceptable PK/PD target attainment. Opportunities to deliver oral narrow-spectrum agents in an optimized format may offer an attractive opportunity within local antimicrobial stewardship agendas and support the avoidance of prolonged courses of IV treatment in certain infections.^6,7^

We explored current and historical literature that compared the use of probenecid with an oral β-lactam antibiotic versus the β-lactam antibiotic alone, describing its impact on PK, clinical outcomes and reported adverse events. The aim was to describe the current literature in support of this approach and identify gaps in knowledge that can be addressed by future mechanistic and efficacy-based research.

## Methods

### Search criteria

We performed a search of MEDLINE and Embase using the search terms outlined in Table S1, available as Supplementary data at *JAC* Online. Studies in English reporting direct comparison of probenecid plus an oral β-lactam versus the oral β-lactam alone in human subjects were included. Common oral β-lactam antibiotics used in the UK were selected for inclusion. These were flucloxacillin, penicillin V, amoxicillin, ampicillin, amoxicillin/clavulanate, cefalexin and cefuroxime axetil. Only full-text, original research articles comparing the addition of probenecid with the same oral β-lactam antibiotic were included. Articles were required to describe PK/PD, microbiology or adverse event outcomes to be included. Anything published before December 2021 was included and no prior time limit was set. Studies were excluded if they were not in English, were reviews and letters, compared different antimicrobial agents or routes of delivery, or reported on non-human subjects. This review was registered on the PROSPERO database prior to data extraction (registration number: CRD42021298765).

### Study selection

Specific literature review software (Covidence, Australia) was used. Two authors (T.M.R. and R.C.W.) independently reviewed abstracts and full texts against inclusion and exclusion criteria. Articles that met screening and eligibility checks were carried forward for full-text review. References of published literature were also reviewed to identify further full texts for inclusion.

### Data extraction

Data were extracted by one researcher (T.M.R.), with cross-checking independently performed by a second author (R.C.W. or M.G.). Data extracted included publication details (authors, journal, year of publication), study details (participants, study design, intervention, control, dosing schedules), primary and secondary outcomes (including PK data and/or clinical outcomes) and reported adverse events/toxicity.

### Risk of bias

Risk of bias for individual studies was assessed in line with Cochrane recommendations. For non-randomized studies, the Risk Of Bias in Non-randomized Studies of Interventions (ROBINS-I) assessment tool was used.^8^ For randomized studies, the Risk of Bias for randomized studies 2 (RoB 2) tool was used.^9^ Risk of bias was assessed by two reviewers (T.M.R. and R.C.W.) independently of each other. Where disagreement in domain scoring occurred, a third reviewer assessed the study and differences were discussed to reach consensus.

### Data analysis

Data were analysed descriptively in line with the aims of this review. For a subset of studies comparing treatment failure between probenecid and control groups, meta-analysis was performed using the ‘metabin’ function from the ‘meta’ package (version 4.11-0) in R (version 3.5.1).^9^ Treatment failure was defined in these studies as microbiological failure, with growth of *Neisseria gonorrhoeae* during follow-up visit after treatment and not associated with self-reported history of re-exposure. Study findings were displayed in forest plots demonstrating the relative risk determined using the Mantel–Haenszel method. Heterogeneity was visually assessed using funnel plots and the *I*^2^ statistic. As study quality was expected to be highly variable, an *a priori* decision was made to proceed with meta-analysis as part of the subgroup analysis despite an expected moderate-to-high risk of bias within studies. Bias plots were generated using the ‘robvis’ package in R.^9^

## Results

### Study selection

**Figure 1.**
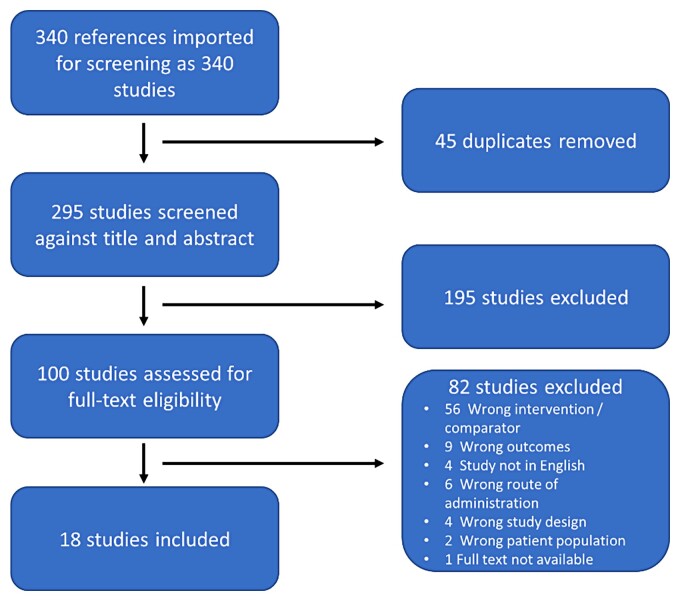
PRISMA diagram summarizing screening and eligibility checking process. This figure appears in colour in the online version of *JAC* and in black and white in the print version of *JAC*.

Figure 1 outlines the study selection process. In total, 340 references were identified, with 45 (13%) duplicates removed. Of the 295 titles and abstracts screened, 100 (34%) were carried forward for full-text review. On full-text review, a further 81/100 (81%) were excluded. Common reasons for exclusion were use of a wrong intervention/comparator agent (56/81; 69%) and wrong outcome measures described (9/81; 11%). One manuscript was not accessible.^10^ Therefore, 18/295 (6%) manuscripts were included in the review.^11–28^

### Study characteristics

Table 1 summarizes studies included. Studies were reported from 1969 to 2021. Populations, methodology and outcome measures were heterogeneous. Most studies were in healthy volunteers^12,14,16,19,20,23–25,27^ (9/18; 50%) or in patients with gonococcal infection^13,15,17,21,22,26^ (6/18; 33%). Additional studies reported on patients with bronchiectasis (1/18; 6%),^11^ biliary pathology (1/18; 6%)^18^ and invasive *Staphylococcus aureus* infection (1/18; 6%).^28^ Crossover trials (11/18; 61%), parallel-arm randomized trials (4/18; 22%), observational (2/18; 11%) and dose-escalation (1/18; 6%) studies were reported.

**Table 1.** Summary of studies comparing the addition of probenecid to an oral β-lactam antibiotic included in the final review

Paper	Population	Design	Intervention	Control	Microbiological outcome	Pharmacokinetic data	Adverse events
Allen *et al.*^11^ 1990	6 patients (4 female) with stable bronchiectasis, median age 53.5 years	Randomized crossover of 3 regimens	Amoxicillin 1 g twice a day plus probenecid 500 mg four times a day OR Amoxicillin 1 g twice a day plus probenecid 1 g twice a day	High-dose amoxicillin 3 g twice a day plus placebo	Nil	Probenecid reduced amoxicillin clearance to one-third of that with the placebo. No influence on *C*_max_ or *t*_½_ identified.	1 patient in probenecid 1 g twice-a-day arm reported nausea
Barbhaiya *et al*.^12^ 1979	8 healthy volunteers, 22–26 years old	Crossover study	Amoxicillin 3 g with 1 g probenecid	Amoxicillin 3 g alone	Nil	Greater peak amoxicillin concentration and larger AUC with probenecid.	N/A
Bro-Jorgensen and Jensen^21^ 1971	1915 men and 921 females with uncomplicated gonorrhoea	Observational study comparing 4 regimens	Ampicillin 1 g plus 1 g probenecid OR Ampicillin 2 g plus 1 g probenecid	Ampicillin 1 g OR Ampicillin 2 g	*Microbiological failure within 14* *days of treatment* Ampicillin 1 g treatment failure: 10.6% in males. Ampicillin 2 g treatment failure: 6.5% in males. Ampicillin plus probenecid failure rate: 1.9% both schedules in males. No significant difference in treatment outcomes in females.		Nil observed
Eriksson^22^ 1973	96 outpatients with uncomplicated gonorrhoea	Observational study	Ampicillin 2 g plus 1 g probenecid	Ampicillin 2 g in divided dose 5 h apart	*Microbiological failure identified during two follow-up visits* Ampicillin plus probenecid treatment failure: 3/24 (13%). Ampicillin: 2/72 (3%), with 3/72 (4%) in this arm also lost to follow-up.	No correlation between serum concentration and recurrent positive culture.	N/A
Everts *et al*.^23^ 2020	11 healthy volunteers (7 female, 4 male)	Crossover study	Flucloxacillin 1000 mg plus probenecid 500 mg	Flucloxacillin 1000 mg	Nil	Probenecid increased the free flucloxacillin AUC and reduced clearance by approximately 53%–55%. 2–5 fold increase in flucloxacillin PK/PD target attainment.	Nil observed
Everts *et al*.^24^ 2021	11 healthy volunteers (7 female, 4 male)	Crossover study	Cefalexin 1 g plus probenecid 500 mg	Cefalexin 1 g	Nil	Probenecid increased cefalexin AUC, *C*_max_ and *t*_½_; enhanced PTA for *S. aureus*.	Nil observed
Frisk *et al*.^25^ 1952	14 healthy volunteers	Dose-escalation study	Penicillin 500 000 units with escalating dose of probenecid from 0.25 mg to 1 g	Penicillin 500 mg alone	Nil	There is a linear relationship between probenecid dose and increase in plasma penicillin concentration in the probenecid dosing range of 0.25–1 g of probenecid.	Nil observed
Gottlieb and Mills^26^ 1986	65 MSM with suspected gonorrhoea	Randomized, parallel-arms study	Cefuroxime 1 g plus probenecid 1 g	Cefuroxime 1 g	*Microbiological failure within 4–7 days of treatment* Probenecid arm had 1/36 failures at 4–7 days; control arm had 3/29 failures.	Nil	N/A
Gower and Dash^27^ 1969	6 healthy volunteers	Crossover study	Cefalexin 1 g four times a day plus probenecid 500 mg four times a day	Cefalexin 1 g four times a day	Nil	Probenecid increased peak cefalexin concentration and serum *t*_½_. Probenecid significantly reduced urinary excretion of cefalexin.	Nil observed
Hedström and Kahlmeter^28^ 1980	6 patients with *S. aureus* infection (4 male, 2 female)	Crossover study	Flucloxacillin 1 g twice a day plus probenecid 1 g twice a day	Flucloxacillin 1 g twice a day	Nil	Probenecid increased flucloxacillin *t*_½_ and doubled AUC in the central compartment.	Nausea and dizziness reported in ‘a few’ patients receiving probenecid 1 g twice a day in a separate observational phase of the study in 35 patients with furunculosis; 1/35 patients reported urticaria and 4/35 exanthem
Karney *et al*.^13^ 1974	155 patients with anogenital gonorrhoea (80 male, 75 female)	Randomized, double-blind, parallel-arms study	Ampicillin 3.5 g plus 1 g probenecid	Ampicillin 3 g	*Microbiological failure within 3–7 days of treatment* Probenecid arm had fewer failures at 14 days, with 1/60 (2%) versus 8/90 (9%) in control arm.	Nil	N/A
Meyers *et al*.^14^ 1969	10 healthy volunteers	Crossover study	Cefalexin 500 mg plus 500 mg probenecid	Cefalexin 500 mg	Nil	Probenecid increased the serum *t*_½_ of cefalexin.	N/A
Mitchell and Robson^15^ 1974	102 males with urethral discharge	Randomized, parallel-arms study	Amoxicillin 2 g plus probenecid 1 g	Amoxicillin 2 g	*Microbiological failure within 28 days of treatment* Cure with probenecid: 50/52 (98%); amoxicillin alone: 39/45 (89%).	Nil	6 patients reported gastrointestinal side effects; unclear whether associated with probenecid arm
Paulsen *et al*.^16^ 1989	12 healthy volunteers (7 male, 5 female)	Randomized crossover study	Amoxicillin 1 g plus probenecid 1 g	Amoxicillin 1 g AND Amoxicillin 3 g	Nil	Probenecid increased amoxicillin *t*_½_ and peak concentration. This led to a doubling of the AUC; no significant difference in PK parameters when compared with 3 g amoxicillin.	N/A
Reichman *et al*.^17^ 1985	124 patients with uncomplicated gonorrhoea (20 female, 104 male)	Blinded, randomized, parallel-arms study	Cefuroxime axetil 1 g plus probenecid 1 g	Cefuroxime axetil 1 g	*Microbiological failure within 4–7 days of treatment* Cure within probenecid arm in 55/56 (98%) versus 50/51 (98%) in control arm.	Nil	Nausea (7/57 versus 1/52) was more predominant with probenecid; vomiting (2/57 versus 1/52) and diarrhoea (6/57 versus 7/52) were similar
Sales *et al*.^18^ 1972	9 patients with T-tubes in the CBD post cholecystectomy	Crossover study	Cefalexin 1 g plus probenecid 500 mg (*n *= 5 patients)	Cefalexin 1 g	Nil	Probenecid led to significant increase in observed bile cefalexin concentration.	N/A
Shanson *et al*.^19^ 1984	10 healthy volunteers	Randomized crossover study	Amoxicillin 3 g plus probenecid 1 g	Amoxicillin 3 g	Nil	Serum concentration was significantly higher at all collected timepoints over 18 h with probenecid.	Nil observed
Staniforth *et al*.^20^ 1983	16 healthy volunteers	Crossover study	Amoxicillin 500 mg plus probenecid 1 g AND Amoxicillin/clavulanate 750 mg plus probenecid 1 g	Amoxicillin 500mg AND Amoxicillin/clavulanate 750 mg	Nil	Probenecid had no effect on clavulanic acid PK; a small change in renal clearance was noted; amoxicillin AUC, *C*_max_ and *t*_½_ were increased.	Nil observed

N/A, not assessed; CBD, common bile duct.

Studies compared different oral β-lactam antibiotics with and without probenecid. These were ampicillin (3/18; 17%), amoxicillin (6/18; 33%), amoxicillin/clavulanate (1/18; 6%), flucloxacillin (2/18; 11%), cefalexin (4/18; 22%), cefuroxime axetil (2/18; 11%) and penicillin V (1/18; 6%). Doses of β-lactam and frequency of treatment varied between study. Most studies described single doses of β-lactam with or without probenecid (15/18; 83%). Probenecid dosing varied between 250 and 1000 mg per single dose in these studies. Primary outcome measures differed between studies, with the effect of probenecid on oral β-lactam PK reported in 12/18 (67%) studies and treatment outcomes (failure of therapy) reported in 6/18 (33%) studies.

### Risk of bias in studies

Figure S1 summarizes the risk of bias for both randomized and non-randomized studies included within this review. Overall, there was a moderate-to-high risk of bias in most studies, with low overall risk in 2/18 (11%) studies only.

### Studies reporting β-lactam PK

Despite variable β-lactam choice and dose, methods of β-lactam quantification and methods of data analysis, common observations were present. Of 12 studies reporting the effect of probenecid on β-lactam PK as a primary outcome, 7/12 (58%) described the influence on AUC, 8/12 (67%) on serum *t*_½_, and 8/12 (67%) on peak observed serum concentration (*C*_max_). Two of 12 studies (17%) reported the use of Monte Carlo simulation to estimate PTA. Addition of probenecid to oral β-lactam antibiotics increased total AUC in 7/7 (100%) studies reporting it. β-Lactam *C*_max_ was significantly increased in 5/8 (63%) and *t*_½_ in 6/8 (75%) of studies reporting these variables. Both studies assessing PTA (2/2; 100%) demonstrated a significant increase in target attainment with the addition of probenecid to β-lactam therapy.

### Studies reporting treatment failure

**Figure 2.**
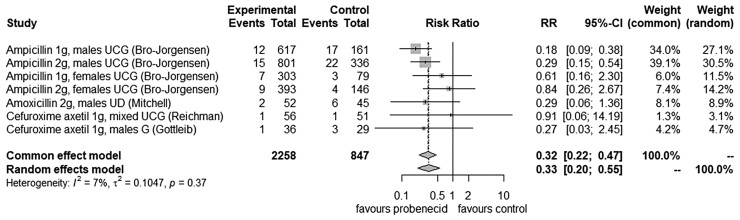
Meta-analysis of the relative risk (RR) of microbiological failure of treatment for gonococcal disease for probenecid-boosted oral β-lactams versus oral β-lactam antibiotic alone. UCG, uncomplicated gonococcal disease; UD, urethral discharge of presumed gonococcal disease; G, gonococcal disease (complicated and uncomplicated); *I*^2^, dispersion of effect size within the meta-analysis; τ^2^, estimated amount of total heterogeneity.

Of the 6/18 (33%) studies reporting on treatment failure as a primary outcome, 4/6 (67%) were included in a meta-analysis comparing the addition of probenecid to an oral β-lactam antibiotic of the same dose on treatment outcome (Figure 2).^15,17,21,26^ One study (17%) could not be included as different doses of ampicillin were used in the intervention and control arms.^13^ A further study (1/6; 17%) could not be included due to different dosing schedules between intervention and control arms.^22^ All four included studies reported on the outcome of treating gonococcal disease, with microbiological failure at follow-up used to define treatment failure. Three (75%) were randomized studies and one (25%) was observational in design. They contained seven direct comparisons of addition of probenecid to an oral β-lactam antibiotic of fixed dose on treatment outcome in 3105 (2258 intervention and 847 control) patients. The relative risk of treatment failure in the random-effects model was 0.33 (95% CI 0.20–0.55; *I*^2 ^= 7%), favouring the addition of probenecid to oral β-lactam regimens.

### Side effects and toxicity

The assessment of side effects/toxicity was reported in 11/18 (61%) studies. Of these, 4/11 (36%) observed side effects, with 7/11 (64%) not reporting any observed adverse events. One randomized study identified a higher rate of reported nausea for 1 g cefuroxime axetil with 1 g probenecid (7/57; 12%) versus 1 g cefuroxime axetil alone (1/52; 2%).^17^ Within this study, rates of vomiting and diarrhoea were similar. A further study highlighted an increase in observed reports of nausea and dizziness associated with 1 g probenecid twice a day in patients receiving 7 days of treatment for furunculosis.^28^ Unfortunately, the observed rate was not quantified by the authors. Allen and colleagues^11^ reported one case of nausea associated with an arm containing 1 g of probenecid twice a day in their study of amoxicillin PK in patients with bronchiectasis. The final study to observe side effects reported six patients with nausea from their entire cohort. The authors do not differentiate between those receiving β-lactam antibiotic alone versus β-lactam antibiotic with probenecid.^15^ PK data for probenecid and/or β-lactam antibiotics were not provided or not available in a way that allowed evaluation of the impact of drug exposure on these reported outcomes.

## Discussion

This review highlights the current paucity of evidence for the use of probenecid to optimize the delivery of oral β-lactam antibiotics. Current data are heterogeneous, use historical methods of drug quantification, and focus predominantly on the management of gonococcal disease. Current evidence suggests that addition of probenecid to oral β-lactam therapy reduces microbiological treatment failures in gonococcal disease compared with use of single doses of an oral β-lactam antibiotic alone. In addition, the influence of probenecid on oral β-lactam PK leads to potentially favourable drug exposures that may enhance target attainment for other infective aetiologies requiring longer courses of antimicrobial therapy, including *S. aureus* infection.

β-Lactam antibiotics exhibit time-dependent mechanisms of action. In the late 20th and early 21st centuries, optimal PK/PD targets for β-lactams have been explored and defined. The time the free (unbound) concentration of β-lactam spends above an organism’s MIC (*fT*_>MIC_) best describes β-lactam PK/PD.^29^ Traditionally, targets of greater than 40%–50% *fT*_>MIC_ are targeted, with evidence that attainment of this target leads to improved patient outcomes.^29^ For some infections, such as those caused by Gram-positive bacteria, lower *fT_>_*_MIC_ may be recommended. However, to prevent the development of *Pseudomonas aeruginosa* drug resistance during therapy, targets between 100% *fT*_>MIC_ and 100% *fT *_> 4–6×MIC_ have been explored.^30–32^ To enhance the efficacy of β-lactam antibiotics, different approaches have been trialled, including prolonged and continuous infusions in patients with variable PK.^33–35^ The benefit of higher doses of oral penicillin for shorter durations has also been demonstrated in conditions such as streptococcal throat infection.^36^ Probenecid’s ability to potentially prolong terminal *t*_½_ and increase *C*_max_ and AUC of both oral and IV agents suggests an alternative option to increasing antimicrobial doses or frequency when optimizing PK/PD targets. Everts and colleagues^23^ demonstrated significant increases in the PTA for the treatment of *S. aureus* using oral flucloxacillin co-administered with probenecid compared with oral flucloxacillin alone in healthy volunteers. These preclinical data are further supported by observational studies reporting favourable outcomes for the management of staphylococcal infections using flucloxacillin with probenecid.^37^ Furthermore, Grayson and colleagues^38^ demonstrated favourable clinical outcomes with IV cefazolin plus probenecid compared with ceftriaxone for the treatment of moderate-to-severe cellulitis as part of a third-generation cephalosporin-sparing regimen.

### Current limitations and future steps

Despite emerging observational data supporting the safety and efficacy of probenecid-boosted oral β-lactam therapy, several mechanistic and efficacy questions remain. Current data are limited by the relatively small sample sizes employed in most studies. No experimental data comparing oral β-lactams with and without probenecid have been reported outside of its use in gonococcal disease. Historical analysis of β-lactam PK often determined total antimicrobial exposure from single drug doses and used old methods of quantification, such as tube dilution methods. These methods were often open to wide variation and make direct comparison between studies challenging. Furthermore, the use of total drug concentration does not allow for the active component (free drug concentration) to be described or understood, meaning that the true impact of probenecid on free antibiotic concentration remains to be defined in many cases. Finally, probenecid is known to interact with a number of common medications seen in multi-morbid patients, including paracetamol, non-steroidal anti-inflammatories, antipsychotic medications and immunosuppressants.^39^ Consideration of these factors on treatment selection and outcomes is lacking from current data.

Future work should focus on characterization of the direct efficacy of addition of probenecid to common oral β-lactam antimicrobial dosing regimens. These studies could include the mechanistic characterization of probenecid’s influence on free chemically active drug and include assessment of clearance, plasma protein binding and target site concentration attainment. As well as demonstrating enhanced antimicrobial PK using probenecid, an impact on antimicrobial PD, clinical outcomes and toxicity must be clearly demonstrated. Future work should include the assessment and definition of probenecid PK/PD. With improved opportunities to provide therapeutic drug monitoring of both oral β-lactams and probenecid,^40,41^ this will further enhance the clinical acceptability of PK manipulation with probenecid and address concerns surrounding potential toxicity, which has not been reported in studies to date.

### Conclusions

Probenecid is associated with improved microbiological cure at follow-up when added to oral β-lactam regimens for the treatment of gonococcal disease. Preclinical and observational data suggest that probenecid-boosted oral β-lactam therapy may have a broader application in the future. To define the potential role of probenecid-boosted oral β-lactam regimens, appropriately powered mechanistic and efficacy-based studies to facilitate direct comparison should be conducted.

## Supplementary Material

dkac200_Supplementary_Data

## Data Availability

Data and materials are available from the authors on reasonable request.

## Supplementary data

Table S1 and Figure S1 are available as Supplementary data at *JAC* Online.
