# Supplementary material for: Addition of probenecid to oral β-lactam antibiotics: a systematic review and meta-analysis
Source: J Antimicrob Chemother. 2022 Jun 21;77(9):2364–72. doi: 10.1093/jac/dkac200 (PMC9721384; doi:10.1093/jac/dkac200)
Supplement: dkac200_Supplementary_Data [file dkac200_supplementary_data.docx]

**Supplementary data**

**Table S1.** Final search terms used

| Query |
| --- |
| Search: (((oral) OR (PO)) AND (penicillin*)) AND ((probenecid) OR (benemid))  ("mouth"[MeSH Terms] OR "mouth"[All Fields] OR "oral"[All Fields] OR ("poisoning"[MeSH Subheading] OR "poisoning"[All Fields] OR "po"[All Fields])) AND "penicillin*"[All Fields] AND ("probenecid"[MeSH Terms] OR "probenecid"[All Fields] OR ("probenecid"[MeSH Terms] OR "probenecid"[All Fields] OR "benemid"[All Fields]))  Translations  oral: "mouth"[MeSH Terms] OR "mouth"[All Fields] OR "oral"[All Fields]  PO: "poisoning"[Subheading] OR "poisoning"[All Fields] OR "po"[All Fields]  probenecid: "probenecid"[MeSH Terms] OR "probenecid"[All Fields]  benemid: "probenecid"[MeSH Terms] OR "probenecid"[All Fields] OR "benemid"[All Fields] |
| Search: (((probenecid) OR (benemid)) AND ((((probenecid) OR (benemid)) AND (amoxicillin)))) AND ((oral) OR (PO)) Sort by: Most Recent  ("probenecid"[MeSH Terms] OR "probenecid"[All Fields] OR ("probenecid"[MeSH Terms] OR "probenecid"[All Fields] OR "benemid"[All Fields])) AND (("probenecid"[MeSH Terms] OR "probenecid"[All Fields] OR ("probenecid"[MeSH Terms] OR "probenecid"[All Fields] OR "benemid"[All Fields])) AND ("amoxicillin"[MeSH Terms] OR "amoxicillin"[All Fields] OR "amoxicilline"[All Fields] OR "amoxicillins"[All Fields])) AND ("mouth"[MeSH Terms] OR "mouth"[All Fields] OR "oral"[All Fields] OR ("poisoning"[MeSH Subheading] OR "poisoning"[All Fields] OR "po"[All Fields]))  Translations  probenecid: "probenecid"[MeSH Terms] OR "probenecid"[All Fields]  benemid: "probenecid"[MeSH Terms] OR "probenecid"[All Fields] OR "benemid"[All Fields]  probenecid: "probenecid"[MeSH Terms] OR "probenecid"[All Fields]  benemid: "probenecid"[MeSH Terms] OR "probenecid"[All Fields] OR "benemid"[All Fields]  amoxicillin: "amoxicillin"[MeSH Terms] OR "amoxicillin"[All Fields] OR "amoxicilline"[All Fields] OR "amoxicillins"[All Fields]  oral: "mouth"[MeSH Terms] OR "mouth"[All Fields] OR "oral"[All Fields]  PO: "poisoning"[Subheading] OR "poisoning"[All Fields] OR "po"[All Fields] |
| Search: ((probenecid) OR (benemid)) AND (cefalexin)  ("probenecid"[MeSH Terms] OR "probenecid"[All Fields] OR ("probenecid"[MeSH Terms] OR "probenecid"[All Fields] OR "benemid"[All Fields])) AND ("cefalexine"[All Fields] OR "cephalexin"[MeSH Terms] OR "cephalexin"[All Fields] OR "cefalexin"[All Fields] OR "cephalexine"[All Fields])  Translations  probenecid: "probenecid"[MeSH Terms] OR "probenecid"[All Fields]  benemid: "probenecid"[MeSH Terms] OR "probenecid"[All Fields] OR "benemid"[All Fields]  cefalexin: "cefalexine"[All Fields] OR "cephalexin"[MeSH Terms] OR "cephalexin"[All Fields] OR "cefalexin"[All Fields] OR "cephalexine"[All Fields] |
| Search: (flucloxacillin) AND ((probenecid) OR (benemid))  ("floxacillin"[MeSH Terms] OR "floxacillin"[All Fields] OR "flucloxacillin"[All Fields]) AND ("probenecid"[MeSH Terms] OR "probenecid"[All Fields] OR ("probenecid"[MeSH Terms] OR "probenecid"[All Fields] OR "benemid"[All Fields]))  Translations  flucloxacillin: "floxacillin"[MeSH Terms] OR "floxacillin"[All Fields] OR "flucloxacillin"[All Fields]  probenecid: "probenecid"[MeSH Terms] OR "probenecid"[All Fields]  benemid: "probenecid"[MeSH Terms] OR "probenecid"[All Fields] OR "benemid"[All Fields] |
| Search: (probenecid) AND (cefuroxime)  ("probenecid"[MeSH Terms] OR "probenecid"[All Fields]) AND ("cefuroxime"[MeSH Terms] OR "cefuroxime"[All Fields] OR "cefuroxim"[All Fields])  Translations  probenecid: "probenecid"[MeSH Terms] OR "probenecid"[All Fields]  cefuroxime: "cefuroxime"[MeSH Terms] OR "cefuroxime"[All Fields] OR "cefuroxim"[All Fields] |

**Figure S1.** Summary of the risk of bias assessment for studies included within the review


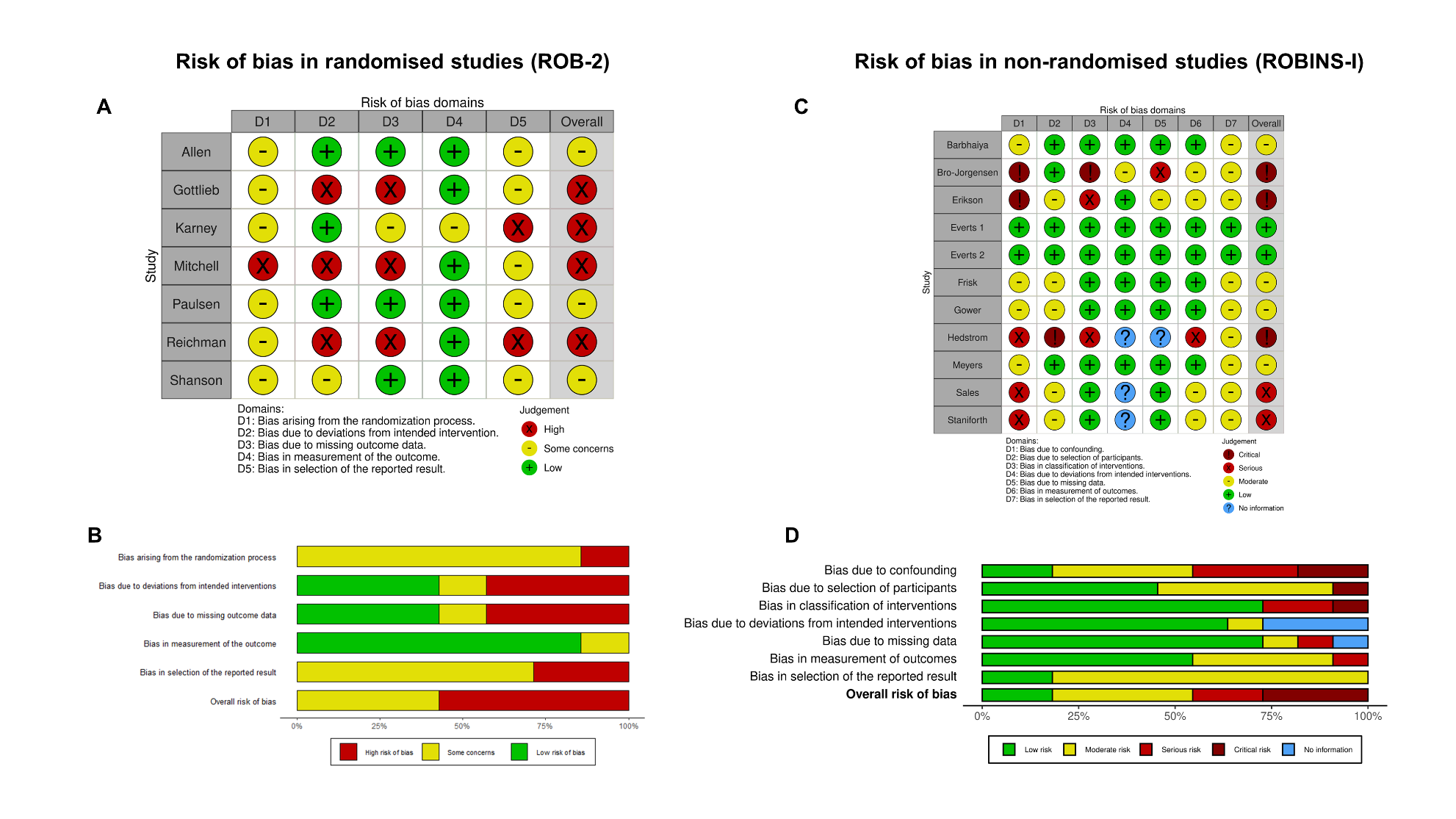


**Legend:**  **A:** Risk of bias domains for individual randomised studies evaluated using the Risk of Bias Assessment Tool version 2 (ROB-2). **B:** Summary of risk of bias domains for randomised studies assessed using ROB-2. **C:** Risk of bias domains for individual non-randomised studies evaluated using the Risk of Bias in Non-randomised Interventional Studies (ROBIN-I) assessment tool. **D:** Summary of risk of bias domains for non-randomised studies assessed using ROBIN-I.
